# Evaluation of the Effectiveness of Insecticide Treated Materials for Household Level Dengue Vector Control

**DOI:** 10.1371/journal.pntd.0000994

**Published:** 2011-03-29

**Authors:** Veerle Vanlerberghe, Elci Villegas, Milagros Oviedo, Alberto Baly, Audrey Lenhart, P. J. McCall, Patrick Van der Stuyft

**Affiliations:** 1 Institute of Tropical Medicine, Antwerp, Belgium; 2 Instituto “Jose Witremundo Torrealba”, Los Andes University, Trujillo, Venezuela; 3 Institute of Tropical Medicine “Pedro Kourí”, Havana, Cuba; 4 Liverpool School of Tropical Medicine, Liverpool, United Kingdom; Centers for Disease Control and Prevention, Puerto Rico, United States of America

## Abstract

**Objective:**

To assess the operational effectiveness of long-lasting insecticide treated materials (ITMs), when used at household level, for the control of *Aedes aegypti* in moderately infested urban and suburban areas.

**Methods:**

In an intervention study, ITMs consisting of curtains and water jar-covers (made from PermaNet) were distributed under routine field conditions in 10 clusters (5 urban and 5 suburban), with over 4000 houses, in Trujillo, Venezuela. Impact of the interventions were determined by comparing pre-and post-intervention measures of the Breteau index (BI, number of positive containers/100 houses) and pupae per person index (PPI), and by comparison with indices from untreated areas of the same municipalities. The effect of ITM coverage was modeled.

**Results:**

At distribution, the proportion of households with ≥1 ITM curtain was 79.7% in urban and 75.2% in suburban clusters, but decreased to 32.3% and 39.0%, respectively, after 18 months. The corresponding figures for the proportion of jars using ITM covers were 34.0% and 50.8% at distribution and 17.0% and 21.0% after 18 months, respectively. Prior to intervention, the BI was 8.5 in urban clusters and 42.4 in suburban clusters, and the PPI was 0.2 and 0.9, respectively. In both urban and suburban clusters, the BI showed a sustained 55% decrease, while no discernable pattern was observed at the municipal level. After controlling for confounding factors, the percentage ITM curtain coverage, but not ITM jar-cover coverage, was significantly associated with both entomological indices (Incidence Rate Ratio = 0.98; 95%CI 0.97–0.99). The IRR implied that ITM curtain coverage of at least 50% was necessary to reduce *A. aegypti* infestation levels by 50%.

**Conclusion:**

Deployment of insecticide treated window curtains in households can result in significant reductions in *A. aegypti* levels when dengue vector infestations are moderate, but the magnitude of the effect depends on the coverage attained, which itself can decline rapidly over time.

## Author Summary

An estimated 40% of the world's population lives at risk of contracting dengue, and it inflicts a significant health, economic and social burden on the populations of endemic areas. In the absence of a vaccine, vector control is the only available strategy to prevent transmission. Some control methods against *Aedes aegypti* (the main dengue vector) have been successful in reducing vector infestation levels, but rarely sustained the reductions for a prolonged period. We report here on the first effectiveness trial of insecticide treated curtains and jar covers against *A. aegypti* implemented under ‘real-life’ conditions. The coverage of tools was high at distribution, but declined quickly over the 18 months of follow up. The vector infestation levels showed a sustained 55% decrease in the intervention clusters, while no discernable pattern was observed at the municipal level. At least 50% curtain coverage was needed to reduce *A. aegypti* infestation levels by 50%. We concluded that deployment of insecticide treated window curtains in households can result in significant reductions in dengue vector levels, which are related to dengue transmission risk. The magnitude of the effect depends on the curtain coverage attained, which itself can decline rapidly over time.

## Introduction

An estimated forty percent of the world's population lives at risk of contracting dengue, which currently is the most important mosquito-borne viral disease worldwide, responsible for 24,000 deaths, 250,000–500,000 hemorrhagic fever cases and up to 50 million dengue infections annually [1], [2]. The public health importance of dengue has grown rapidly in recent years, with a 30-fold increase in incidence since the 1960s. This has coincided with the expansion of the geographical range of its main vector, the mosquito *Aedes aegypti* [2], [3], and co-circulation of multiple dengue serotypes, which elevates the risk of sequential infections and severe disease [4].

No curative treatment is available and the prevention of a fatal outcome in severe dengue cases hinges on early case detection and appropriate supportive treatment. To decrease the burden of disease, prevention of transmission is crucial. As there is no vaccine yet, this is possible only by vector control. Existing *A. aegypti* control tools can reduce vector infestation levels, but very few have succeeded in sustaining reductions for a prolonged period [5], [6] or in impacting on dengue transmission [7], [8]. The national routine dengue vector control programmes in endemic countries are facing variable and often disappointing results, which are among others due to inadequate implementation processes, lack of community participation or poor user acceptance of chemical-based vector control methods [9], [10]. Programs integrating chemical or biological based strategies with community involvement are having better results, but rarely eliminate the vector [11]–[14], though there have been notable successes in recent years [8].

Insecticide treated materials (ITMs) have recently shown promise in reducing household level dengue vector infestations [15]–[17]. Unlike most dengue vector control strategies, ITMs target the adult mosquito, which is epidemiologically the most important vector stage. It is postulated that the likelihood of adult vectors contacting an ITM during host seeking reduces their life expectancy, effectively altering the age structure of the vector population, such that fewer mosquitoes live long enough to become infective with dengue [9]. Furthermore, ITMs made from long-lasting insecticide treated fabrics retain their efficacy for at least 1 year [18], which is longer than any other applied *Aedes* control tool. In previous trials, ITMs were shown to have an impact on vector populations and to have high acceptance levels by householders up to a few months after distribution [15], [17], though the key question of whether or not this will result in reduced dengue transmission remains to be proven before ITMs can be recommended as dengue vector control tools on a large scale. Achieving and sustaining high levels of ITM uptake and use under routine programme conditions rather than in an experimental situation also are fundamental prerequisites to success and this too requires investigation.

We report here on an intervention study in urban and suburban areas of Trujillo State, Venezuela, where insecticide treated window curtains and water jar covers were distributed by local health committees and by the existing routine vector control programme. Over a period of 18 months, we assessed the uptake and use of these tools by local householders and their effectiveness in controlling the vector population, comparing the Breteau and the pupae per person indices at several time points before and after intervention; and also comparing them with indices from untreated areas of the same municipalities.

## Materials and Methods

### Ethics Statement

This study received clearance from the ethical committee that oversees research of the Institute of Tropical Medicine, Antwerp and from the bio-ethics committee of the Jose Witremundo Torrealba Research Institute, Trujillo. Community representatives from each participating cluster approved the intervention and written informed consent was obtained from each individual household included in the study. The ITMs were made from material that is approved by the World Health Organization Pesticide Evaluation Scheme (WHOPES) for bed net use. The trial was registered at ClinicalTrials.gov (number NCT 00883441).

### Study setting

The study was conducted in the municipalities of Valera (9°19′N 70°36′W; altitude 541 m) and San Rafael de Carvajal (9°20′N 70°35′W; altitude 556 m; referred to as Carvajal) in Trujillo State in north west Venezuela. The climate is tropical with two rainy seasons (March/April and September/November), an average annual rainfall of 750 mm and temperatures ranging from 16–37°C. The city of Valera is the economic capital of the state, with 128,556 inhabitants and a population density of 534 inhabitants/km^2^. Carvajal is a suburban municipality located 4 km from Valera, with 44,213 inhabitants and a population density of 493 inhabitants/km^2^.

Dengue is endemic in Trujillo State. Between 2006 and 2008, dengue case reports ranged between 203 and 396 cases/100,000 inhabitants/year, of which 1.1 to 4.9% were hemorrhagic cases (Regional direction of epidemiology and statistics, Trujillo state health ministry). In Trujillo, dengue affects children and young adults (up to 24 years old) primarily, with cases peaking between August and December.

All routine *A. aegypti* vector control activities are carried out by a team of 24 persons from the department of environmental health of the Trujillo state health ministry. Activities comprise adulticiding (indoor spraying with malathion 94% ULV) and larviciding (Abate) within a 200 meter radius of a reported dengue case. When the number of clinical cases exceeds the epidemic alert level of the endemic channel, space spraying with vehicle-mounted equipment is added (Department of environmental health of the Trujillo state Health Ministry).

### Study design

The study had been designed as a cluster randomized trial for comparing the efficiency of 2 ITM distribution models in terms of uptake and continued use that would at the same time permit a non-randomized before - after comparison to evaluate the effectiveness of the ITMs. Uptake rates attained at distribution and the subsequent reductions in coverage over time were equivalent in both models and are not analyzed in depth in this manuscript.

In March 2006 10 clusters (defined as distinct neighbourhoods of 300–600 houses) were recruited for an intervention study and were stratified based on location in urban or suburban areas, which differ in *Aedes* infestation levels and population characteristics. The 10 clusters were selected from 18 districts that had dengue notification rates of at least 40/10,000 inhabitants (2003–2005). The inclusion criteria at cluster level were: middle or low socio-economic status (the number of high socio-economic level clusters was small and they were not representative of the overall area) with fewer than 50% of the population residing in apartment blocks (for operational reasons). Rural areas, where principal land use was for agricultural activities and where dengue was not a major health problem, were excluded.

The sample size (number of clusters) was determined using calculations proposed by Hayes and Bennett [19], and had a power of 80% to detect a 5-fold decrease in the Breteau index at an alpha error level of 0.05 (assuming a between-cluster coefficient of variation of 0.50).

### Intervention

After collecting baseline data for 1 year, insecticide treated (IT) curtains and IT jar covers were distributed to all households in the 10 intervention clusters that had given their informed consent and agreed to use them. This was done between July and September 2007 either by the routine vector control programme or by local health committees. The ITcurtains and ITcovers were made from the same PermaNet (Vestergaard-Frandsen) polyester netting treated with a long-lasting formulation of deltamethrin (55 mg/m^2^), coated with an unknown protectant (not disclosed by the manufacturer) to prevent degradation of the insecticide when exposed to UV light. The manufacturer stated that this material does not require re-treatment and its insecticidal effect is expected to last for up to 2 years or 6 “standard” washes (http://www.vestergaard-frandsen.com/permanet-curtain-e-brochure.pdf, accessed 22/05/2008). The number of ITcurtains and ITcovers distributed per house depended on the number of windows in the main living area and bedrooms (up to a maximum of 5 curtains/house) and on the numbers of 150–200 liter water storage jars present in the house (no maximum of covers per house).

During distribution, at least one person in every household received information on the use and maintenance of the ITMs through person-to-person communication.

### Data collection

A total of 1120 houses were selected through systematic random sampling (560 houses across all urban clusters, corresponding to every 3^rd^ house; and 560 across all suburban clusters, corresponding to every 4^th^ house) for periodic entomological monitoring. Five independent entomological surveys were conducted by a survey team (trained and supervised by an experienced entomologist, author MO) at roughly six-month intervals: two pre-intervention surveys (October 2006, March/April 2007) and three post-distribution surveys (November/December 2007, April 2008, January 2009). In all houses, containers were inspected for the presence of larvae and pupae. If pupae were found they were counted, collected, transported to the laboratory and allowed to emerge for species identification.

As external control data, in line with Kroeger *et al.* [15], we used the entomological data collected in the municipalities of Valera and Carvajal as part of the routine surveillance activities. The intervened clusters represented 7% of the total number of houses in the municipalities and the populations of vectors in the municipalities were considered beyond the influence of the ITMs used in the study clusters, and expected to fluctuate naturally as influenced by seasonal parameters only. The routine entomological surveillance data were collected by the department of environmental health of the Trujillo state health ministry: Houses in a radius of 200 m around a confirmed dengue case are visited and infestation of water holding containers with immature vector stages is recorded. We report the routine data for the months when entomological surveys were conducted in the intervention clusters and correct for the differences in data collection methods in the analyses (see subsection on data analysis).

Rainfall and temperature data were obtained from the weather station at the Valera airport (located in between the two study sites, that are, themselves, 4 km apart) (http://www.wunderground.com/history/station/80426). The averaged rainfall data from the month of each entomological survey plus data from the preceding month were used in our analyses. Data on routine *A. aegypti* control activities that took place in the intervention clusters during the month of each entomological survey and the preceding month were retrieved from the reports of the vector control programme. For each cluster, an intensity score was calculated based on the number of houses treated per cluster (adulticiding and/or larviciding): 0 = no activities, 1 = less than 10% of houses covered, 2 = between 11 and 20% of houses covered, 3 = between 21 and 50% houses covered, 4 = more than 51% of houses covered, 5 = intensive and repeated spraying and larviciding in all houses.

A baseline socio-economic survey was conducted during June–July 2006 in a systematic random sampling of 955 households (465 urban and 490 suburban). The survey encompassed both general and dengue related household characteristics. We used the Graffar Method, adapted to the Venezuelan context by Méndez-Castellano [20] to classify the households according to socio-economic stratum based on the profession of the head of household, education level of the mother, main source of family income and housing conditions. It classifies households from stratum I (upper class) to stratum V (critical poverty). A random sub-sample of households that participated in the baseline sociological survey was revisited in September 2007, February 2008 and January 2009 to observe the presence and use of the ITMs. On the same occasions, we inquired about any adverse effects attributed to the use of the ITMs.

### Data analysis

We developed 2 indicators for assessing ITcurtain coverage per cluster: the percentage of houses with at least 1 curtain and the median number of curtains per house. For ITcovers, the 2 indicators were the percentage of houses using at least 1 ITcover and the percentage of eligible water storage jars covered. We used the chi square test and the Mann-Whitney test to compare urban and suburban coverage proportions and medians respectively, and calculated 95% confidence intervals for their differences.

*A. aegypti* infestation levels were the outcome measures. We calculated the Breteau index (BI, number of containers positive with immature *A. aegypti*/100 inspected houses) per cluster, per setting (urban/suburban) and per survey round. We compared trends over time for the BI in the urban and suburban study clusters. The trend of the BI in the corresponding municipalities was used to control for the natural seasonal fluctuations in vector populations. We calculated the % difference between the values at each survey time point and the October 2006 pre-intervention values. This permitted to represent the trends and to allow, at the same time, for the different methodology used to measure BI in intervention clusters and the municipality. For the intervention clusters, the pupae per person index (PPI, number of *A. aegypti* pupae/inhabitant) - considered a more accurate proxy for adult mosquito abundance [21] - was also calculated per cluster, per setting and per survey round. 95% confidence intervals around each estimate at each time point were calculated with a negative binomial regression model taking into account the cluster design.

To estimate the independent effect of ITM coverage on *A. aegypti* infestation at the cluster level, we constructed two generalized linear random effect regression models with a negative binomial link function, taking into account the repeated measurements. Both outcome measures, BI and PPI, were the dependent variables. Each of the 10 clusters contributed 1 data point at each of the 5 entomological survey rounds. The models included the % ITcurtain and ITcover coverage, the setting (urban or suburban), intensity of routine vector control activities in each cluster, municipal level data on *A. aegypti* infestation, rainfall and temperature. Interaction between variables was assessed. Based on the corresponding model regression-coefficient, the independent effect of ITcurtains coverage on BI was graphically represented over the empirically observed coverage range.

Data were analyzed with Stata 10.0 (StataCorp, Texas, USA) and SPSS 17.0 (SPSS Inc., Chicago, IL, USA). In less than 1% of cases some essential data were missing, and these households were subsequently withdrawn from the database.

## Results

The 5 urban and the 5 suburban clusters contained 1742 and 2359 houses, respectively. All clusters completed the study protocol through January 2009 and all were included in the analysis. Households in urban clusters had a significantly higher socio-economic status (p<0.05) (Table 1). Permanent water supply was more common in urban than suburban intervention clusters (65.8% and 44.9% respectively; p<0.001), while houses in suburban clusters had more water storage jars than urban houses (averages of 1.2 and 0.5 water storage jars/house respectively; p<0.05).

**Table 1:** Baseline characteristics of the study site, classified as either urban or suburban clusters. Trujillo State, Venezuela, July 2006.

	Urban area(number and %)	Suburban area(number and %)
**Socio-economic level** 1		
Stratum I (highest level)	11 (2.4)	10 (2.0)
Stratum II	71 (15.3)	51 (12.8)
Stratum III	151 (32.5)	123 (25.1)
Stratum IV	227 (48.8)	282 (57.6)
Stratum V (lowest level)	5 (1.1)	24 (4.9)
Frequency of water supply		
Permanent	306 (65.8)	220 (44.9)
Irregular, every 2 to 7 days	118 (25.4)	265 (54.1)
Irregular, every 8 days or more	41 (8.8)	5 (1.0)
Mean number of water storage jars/house (SE)	0.5 (0.4)	1.2 (0.3)
Proportion of jars fitted with covers^2^	73.1%	35.9%
Applying larvicides^3^	43 (9.2)	41 (8.4)
Past history of dengue in family^3^	154 (33.1)	123 (25.1)

1 *As determined by the Graffar method (see* *Materials and Methods* *section).*

2 *Non-insecticidal covers bought locally and fitted prior to the study.*

3 *As reported by Households.*

All 510 pupae collected in the October 2006 survey belonged to subgenus Stegomyia and 89% were *A. aegypti*. Also, in November 2007, after ITM distribution, 89% of the collected pupae were identified as *A. aegypti*. Since the vast majority of immature stages were *A. aegypti* at both time moments, all immature stages observed in entomological surveys were assumed to be of this species, in line with Arredondo-Jimenez and Valdez-Delgado [22].

Immediately after ITM distribution, in September 2007, coverage with ITcurtains was similarly high (>75%) in both settings (Table 2). ITcover coverage levels differed significantly between settings, with lower coverage in urban (12.9% of houses) compared to suburban (31.1%) clusters (p<0.001). This was not surprising since, at baseline, 73.1% of jars in urban clusters were found to be fitted with a locally purchased cover as compared to 35.9% in suburban clusters (regardless of condition or use)(p<0.05).

**Table 2:** Coverage with insecticide treated materials at distribution in the intervention clusters. September 2007, Trujillo State, Venezuela.

	Urban	Suburban	Difference(95% CI)	p-value
**IT Curtains**				
% houses with ≥1 curtain	79.7%(366/459)	75.2%(375/499)	4.5%(1.8%–7.2%)	0.09
Median n° curtains per house (P25–P75)	3(2–5)	3(1–5)	0	0.04*
**IT Jar covers**				
% houses with ≥1 cover	12.9%(59/459)	31.1%(155/499)	−18.0%(−20.6%–−15.4%)	0.000*
% jars covered with ITcover	34.0%(87/256)	50.8%(272/535)	−16.8%(−20.5%–−13.1%)	0.000*

*denotes significant difference.

Minor allergic reactions (temporary [less than 48 hrs] itching of palms) after handling the ITMs were reported in 5.4% of houses.

**Figure 1:**
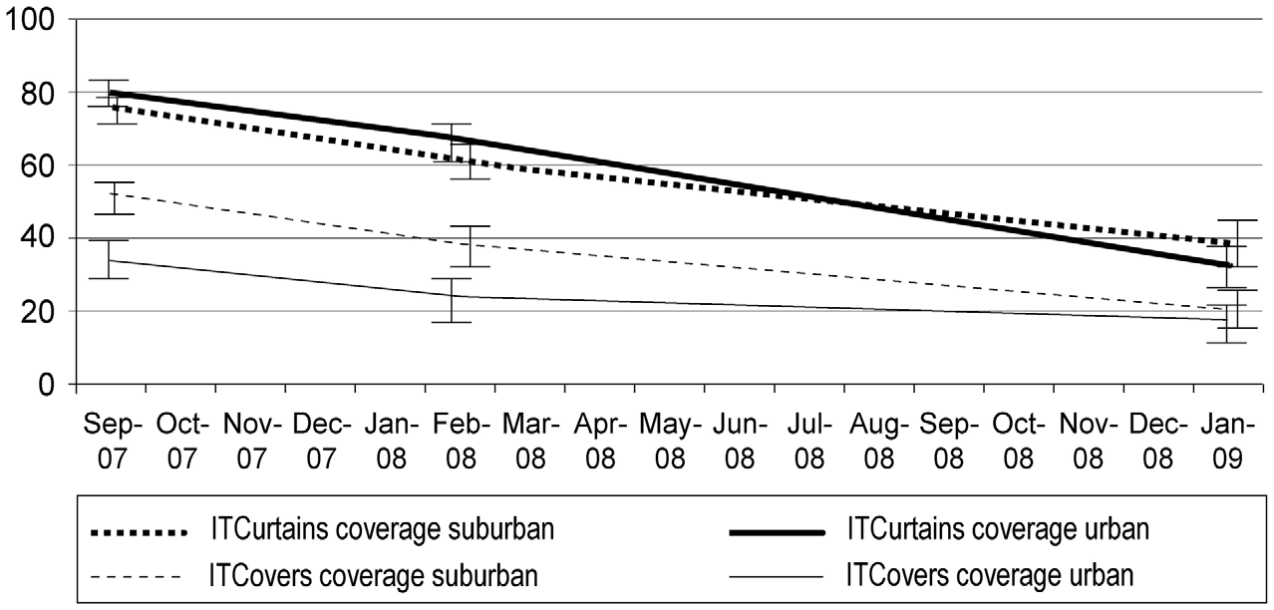
Coverage with Insecticide Treated Materials over time (%; 95% CI), Venezuela, 2007-2009. *Bold dashed line:* IT curtain coverage (% of houses with ≥ 1 ITcurtain) suburban setting. *Dashed line:* IT jar cover coverage (% of jars with ITcover) suburban setting. *Bold solid line:* IT curtain coverage (% of houses with ≥ 1 ITcurtain) urban setting. *Solid line:* IT jar cover coverage (% of jars with ITcover) urban setting.

As the trial progressed, ITM coverage declined such that by the end of the 18-month follow-up period (January 2009), fewer than 40% of houses were using ITcurtains and fewer than 20% were using ITcovers (Figure 1). There were no significant differences between the settings (urban or suburban) in the rates of decline in coverage of ITcurtains (p>0.05) or ITcovers (p>0.05). It was observed that the ITcovers deteriorated as the study progressed, particularly the elasticated rim, resulting in poorly sealed jars.

**Figure 2:**
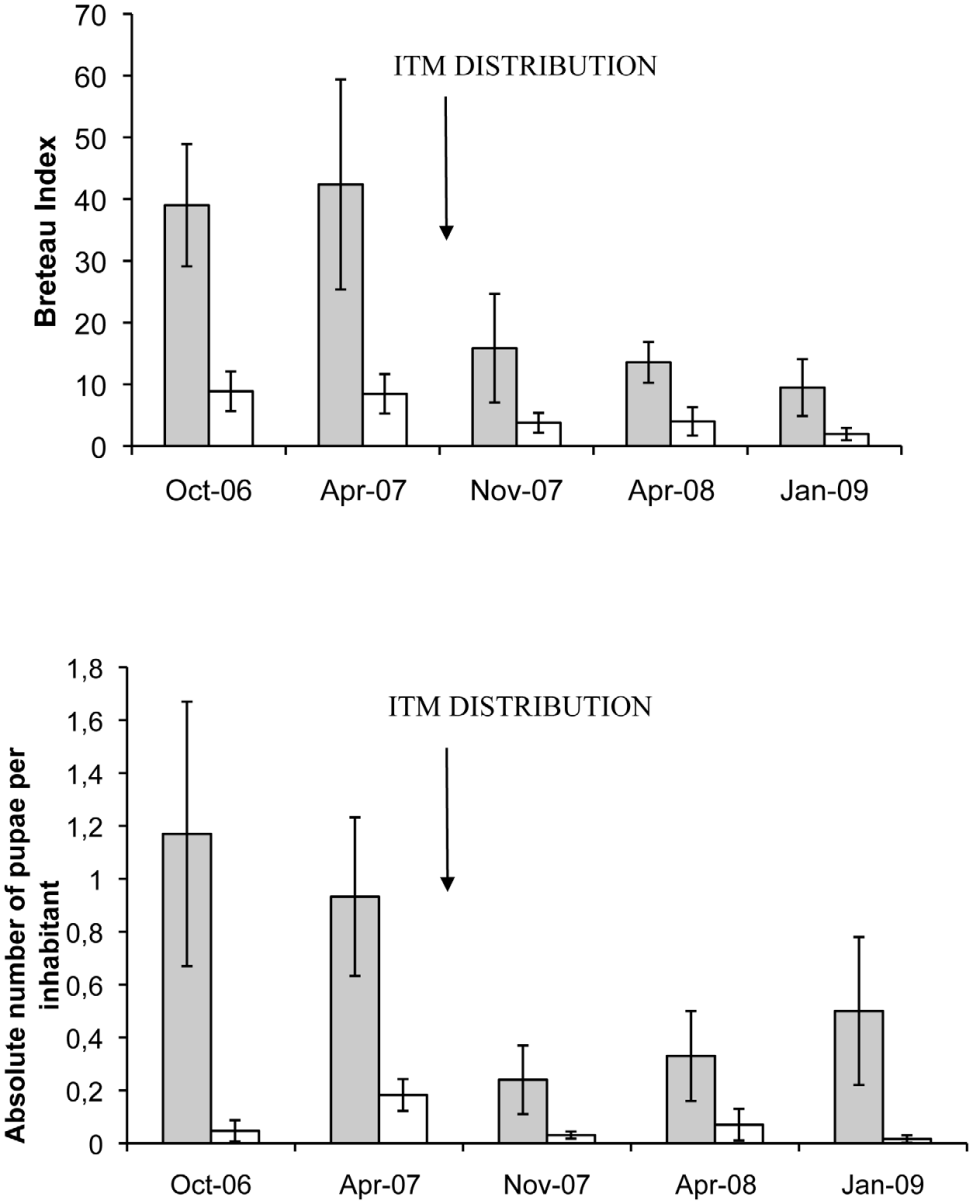
Breteau indices and pupae per person indices in the intervention clusters (with 95% CI), Venezuela, 2006-2009. *Shaded box:* Suburban setting. *White box:* Urban setting. Breteau index: number of positive containers/100 houses; Pupae per person index: number of *A. aegypti* pupae/inhabitant.

**Figure 3:**
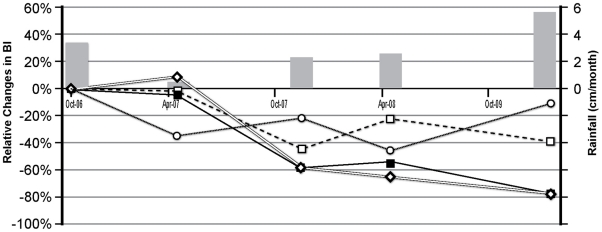
Rainfall and Relative changes in Breteau indices in intervention clusters and municipalities before and after ITM introduction, Venezuela, 2006-2009. *Dashed line:* Urban municipality. *Solid line:* Urban intervention clusters. *Dotted line:* Suburban municipality. *Double line:* Suburban intervention clusters. *Shaded box:* Rainfall. Breteau index: number of positive containers/100 houses.

Prior to intervention, the BI was 42.4 in suburban intervention clusters, which was significantly higher than the BI of 8.5 in the urban clusters; the PPI was 0.9 and 0.2, respectively (Figure 2). Both settings experienced significant declines in the BI (IRR = 0.30, 95% CI 0.22–0.49) and PPI (IRR = 0.23, 95%CI 0.14–0.37) in the months following the distribution of the ITMs (Figure 2). In November 2007, the BI fell to 15.8 in urban and 3.8 in suburban intervention settings, and the PPI decreased to 0.2 and 0.03, respectively. While the PPI gradually increased again in the suburban (but not in the urban) clusters, the BI remained consistently at 55% or more below pre intervention levels in both settings throughout the 18-month follow up period. In contrast, BI levels in urban and suburban municipalities (59.0 and 82.6 respectively in October 2006) fluctuated considerably and did not show the same patterns as the study areas (Figure 3). The differences in average BI between the pre- and post-intervention period was −63% for urban and −67% for suburban intervention clusters. For the corresponding municipal areas these differences were −35% and −26% respectively.

**Figure 4:**
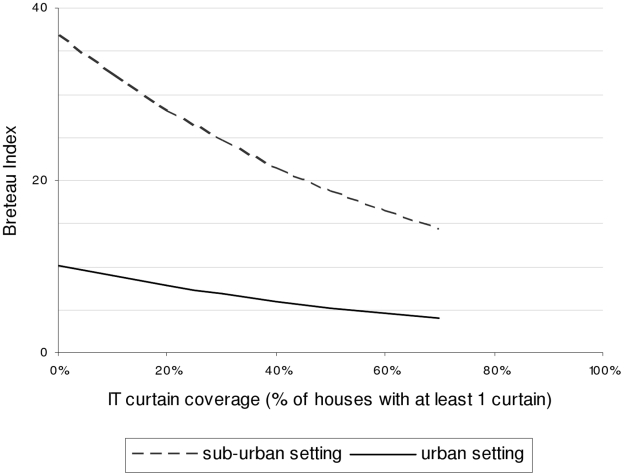
Modeled independent effect of ITcurtain coverage on the Breteau index, Trujillo State, Venezuela, 2006-2009. *Dashed line:* Suburban setting. *Full line:* Urban setting.

In the random effects negative binomial regression models (Table 3), the setting (urban or suburban) and the amount of rainfall were significantly correlated with the BI and PPI in the intervention clusters. Overall infestation levels at the municipality level and temperature had no significant effect, and it was interesting to note that the intensity of routine *A. aegypti* control activities in the clusters also had no effect. Temperature was not included in the final model because it did not confound the relationship between BI or PPI and ITcurtain coverage. ITcurtain coverage was highly significantly correlated with both entomological indicators, but ITcover coverage was not. Each 1% coverage increase with ITcurtains reduced the BI and PPI by 2%. Plotting this effect (Figure 4), reveals that 50% coverage or more was needed to halve the BI, and that the reduction, in absolute terms, depended on the initial BI.

**Table 3:** Independent determinants of household *A.aegypti* infestation rates in the intervention clusters. Trujillo State, Venezuela, 2006–2009.

	Breteau index		Pupae per person index	
	Incidence Rate Ratio (95% CI)**	p-value**	Incidence Rate Ratio (95% CI)**	p-value**
ITCurtain coverage (%)	0.98 (0.97–0.99)	0.008*	0.98 (0.97–0.99)	0.012*
ITcover coverage (%)	1.00 (0.99–1.02)	0.519	1.01 (0.99–1.01)	0.395
Suburban setting	2.88 (1.73–4.78)	0.000*	3.80 (1.93–7.46)	0.000*
Intensity of routine *Aedes* control activities	0.96 (0.85–1.08)	0.473	0.87 (0.73–1.03)	0.100
Rainfall (cm/month)	0.82 (0.73–0.91)	0.000*	0.87 (0.75–1.01)	0.065
*Aedes* infestation at municipality level	1.01 (0.99–1.03)	0.139	1.01 (0.99–1.03)	0.363

*significant at <0.05 level.

**Random effects negative binomial regression model. BI and PPI measured at 6 month intervals at the cluster level.

*No significant interaction terms.*

## Discussion

The presence of insecticide treated window curtains in an environment where *A. aegypti* infestation levels are moderate (BI ranging between 10 and 50) can lead to substantial reductions in the Breteau index and the pupae per person index. The scale of the effect depends on the household coverage attained, and without any further intervention, curtain usage may rapidly decline over time. The demonstration of an effect on the PPI, in addition to the BI, is important, as PPI is considered a more accurate measure of local adult vector abundance, and therefore more directly related to dengue transmission risk [21].

We were unable to directly monitor adult *A. aegypti* populations (due to operational reasons and resource constraints) let be to measure dengue transmission. This study was also limited by the fact that it is a before and after evaluation and that we did not include randomized control clusters in the design, but used routine entomological surveillance data from the whole municipality as ‘control’ data. However, it is not likely that temporal trends in vector density should selectively affect the intervention clusters only and bias, if any, could not explain the differences that we observed. Importantly, the effects we attribute to the actual coverage with ITcurtains and ITcovers are independent of the comparison with the control data at specific time points, and of possible confounding factors such as rainfall, temperature, routine vector control activities and temporal trends that were controlled for in the analysis. Additionally, it has been demonstrated that the insecticide in the PermaNet curtains, when used by households, remains effective for at least 1 year [18]. Furthermore, the local mosquito population remained susceptible to deltamethrin, as shown in bioassays on *A. aegypti* collected in neighbouring municipalities where the same ITcurtains were concurrently deployed in the frame of another study (A. Lenhart, personal communication).

Major strengths of this study, from a public health perspective, are the length of the pre- and post intervention data collection periods and the fact that the ITMs were introduced into the community by the routine vector control programme and the local health committees, which mimics the reality of routine operational conditions. Both these elements markedly distinguish our approach from the one used in the only previous study on ITcurtain deployment for dengue control [15]. In that study, Kroeger *et al.* [15] reported no differences in entomological indices between intervention and control arms. This lack of a difference was attributed to a “spill-over” of the effect of the IT curtains in the intervention clusters into the adjacent control clusters. The authors performed therefore a before and after evaluation of Stegomyia indices, using routine surveillance data collected in nearby communities, and concluded that the changes in Stegomyia indices in the control and intervention study areas combined, could not be explained by natural fluctuations in the vector population due to seasonal parameters.

The coverage attained at ITcurtain distribution in our study is lower, but still comparable to the 87% coverage attained in the Venezuelan site of the above efficacy trial [15], in which delivery was controlled by the research team, and we achieved reductions in vector density of the same order of magnitude. However, our follow up period was much longer and our results on sustained use differ markedly. While coverage remained stable up to the final observation at month 5 in Kroeger *et al.* [15], we observed a 20% decline in use 6 months after distribution and over 50% at 18 months. The determinants of uptake and continued use of the ITM have been assessed [23] and it was found that uptake was linked to pre-use behaviour and contextual factors, but continued use was mainly determined by the perceived effectiveness of the tool. Furthermore, our results demonstrate that the level of coverage attained has profound implications for the effectiveness of ITcurtain interventions.

We did not find a significant effect of ITcovers on entomological indices. Kroeger *et al.* [15] reported on the combined efficacy of ITcurtains and ITcovers, but did not study ITcover efficacy independently. They also remarked that the covers were not durable and were easily torn and that use decreased by over 30% in the initial 5 months, which concurs with our own observations. In contrast, a trial of ITcovers in Cambodia [17] reported a much higher coverage of 3.1 ITcovers per house and a 58% reduction in PPI at 13 weeks in the intervention area as compared to the control area (dropping to 13% at 22 weeks post-intervention). Inherent differences between the studies, in *A. aegypti* oviposition behavior and/or variations in ITcover coverage or the quality of the materials used might explain the differences in results between studies, but further research is needed to clarify exactly how ITcovers impact on dengue vector populations.

Comparisons of our results with those obtained in controlled trials of other dengue vector control tools are difficult, certainly at quantitative level, as there are variations in study design, follow-up periods and/or outcomes measured. Although direct comparisons cannot be made, a number of issues are noteworthy. First, apart from the promising results observed with insecticide treated materials [15]–[17], and small field-laboratory studies of lethal ovitraps, (*e.g.* in Brazil; [24]), all published controlled intervention studies to date have targeted immature dengue vector stages. Secondly, substantial and sometimes sustainable effects have been reported with approaches combining chemical and biological control [25], chemical control and community based environmental management [13], [14], [26] , and biological control and environmental management [27]. Hence, strategies integrating multiple control measures appear to be more effective [28]. Thirdly, only very few of the control strategies managed to completely eliminate the vector [8].

Against this backdrop, we cautiously state that ITcurtains constitute a potentially effective novel tool for controlling *A. aegypti*, with efficacy likely to be optimized when deployed in combination with other vector control tools, and particularly when their use is embedded in a strategy that also mobilizes the community. However, before calling for the launch of large scale integrated effectiveness trials with ITcurtains, important questions remain regarding the efficacy, cost and implementation of ITM strategies: Does long lasting material remain effective beyond one year [18] when heavily exposed to sunlight and dust? How efficacious are ITMs at low or very high *A. aegypti* infestation levels and, ultimately, what is their impact on dengue transmission? What is their incremental cost-effectiveness at 0.93 USD per m^2^ of fabric plus approximately 0.5 USD for distribution [29]? And, obviously, finally, how can the high level of coverage required for effectiveness be attained and maintained under routine conditions?
